# Evolution and Genetic Diversity of Porcine Circovirus 3 in China

**DOI:** 10.3390/v11090786

**Published:** 2019-08-27

**Authors:** Ye Chen, Quanming Xu, Hong Chen, Xian Luo, Qi Wu, Chen Tan, Qidong Pan, Ji-Long Chen

**Affiliations:** 1 Key Laboratory of Fujian-Taiwan Animal Pathogen Biology, College of Animal Sciences, Fujian Agriculture and Forestry University, Fuzhou 350002, China; 2 Fujian Agriculture and Forestry University, Fuzhou 350002, China; 3 CAS Key Laboratory of Pathogenic Microbiology and Immunology, Institute of Microbiology, Chinese Academy of Sciences, Beijing 100101, China

**Keywords:** PCV3, emerging, evolution, genetic diversity, evolutionary dynamics

## Abstract

The identification of a new circovirus (Porcine Circovirus 3, PCV3) has raised concern because its impact on swine health is not fully known. In Fujian Province in eastern China, even its circulating status and genetic characteristics are unclear. Here, we tested 127 tissue samples from swine from Fujian Province that presented respiratory symptoms. All of the PCV3 positive samples were negative for many other pathogens involved in respiratory diseases like PCV2, PRRSV, and CSFV, suggesting that PCV3 is potentially pathogenic. From phylogenetic analysis, PCV3 strains are divided into two main clades and five sub-clades; PCV3a-1, PCV3a-2, PCV3a-3, PCV3b-1, and PCV3b-2. Our identified strains belong to genotypes PCV3a-1, PCV3a-2, PCV3a-3, and PCV3b-2, indicating a high degree of genetic diversity of PCV3 in Fujian province until 2019. Interestingly, we found the time of the most recent common ancestor (tMRCA) of PCV3 was dated to the 1950s, and PCV3 has a similar evolutionary rate as PCV2 (the main epidemic genotypes PCV2b and PCV2d). In addition, positive selection sites N56D/S and S77T/N on the capsid gene are located on the PCV3 antigen epitope, indicating that PCV3 is gradually adaptive in swine. In summary, our results provide important insights into the epidemiology of PCV3.

## 1. Introduction

The *Circovirus* genus claimed veterinarians’ attention shortly after its discovery, most members infect avian species but swine also serve as natural hosts [1,2]. PCV1 and PCV2 are infectious agents in swine [3]; PCV1 was first detected as a contaminant of PK-15 cell cultures and has not been associated with clinical disease, PCV2 is a ubiquitous, economically important pathogen to the swine industry [4]. PCV2 infection leads to a diverse range of clinical diseases collectively termed PCV2-associated disease (PCVAD), which includes post-weaning multi-systemic wasting syndrome (PMWS), porcine dermatitis and nephropathy syndrome (PDNS), interstitial pneumonia, enteric disease, and reproductive failure [5]. Porcine Circovirus 3 (PCV3) is the third member of the *Circovirus* genus to infect swine [6], it has a circular ssDNA genome of approximatively 2000 bases containing three identified ORFs [6], although only ORF1 and ORF2 have been characterized. ORF1, located on the positive strand, apparently codes for a single replication-associated protein (Rep) of 296–297 aa [6]. ORF2 is on the negative strand and encodes the capsid protein (Cap). Despite the common genomic organization, PCV3 is distantly related to other known circoviruses, although a relatively close relationship with bat circoviruses has been suggested based on phylogenetic analysis [7]. There is also some unresolved opinion on the genotyping of PCV3. For example, Li et al. divide PCV3 into 3a and 3b, while Ouyang et al. suggest that PCV3 can be divided into PCV3a, 3b and 3c [7,8].

PCV3 was first identified in the USA in 2015; from tissues of animals presenting porcine dermatitis and nephropathy syndrome (PNDS) and reproductive disorders, but that had tested negative for PCV2; a metagenomics approach revealed an uncharacterized virus [6]. PCV3 has since been identified in Asia [9,10,11], South America, and Europe [12,13,14,15,16] in swine with various clinical syndromes [6]. Its comparable prevalence in healthy swine [17] and wild boar [18,19] however, raises questions about its role in swine pathogenesis.

In China, the presence of PCV3 has been confirmed in more than 24 provinces and regions, but its prevalence on swine farms (especially in asymptomatic herds) is not well established and specific genetic information is lacking, particularly in eastern China. The aim of this study was to determine the infection status and genetic characteristics of PCV3 in swine presenting respiratory symptoms on farms in Fujian province, eastern China. In addition, we aimed to elucidate the evolution and genetic diversity of PCV3 at the national scale.

## 2. Material and Methods

### 2.1. Tissue Samples, DNA Extraction, PCR Amplification, and Sequencing

One hundred and twenty-seven lung samples were collected from one hundred and twenty-seven swine on fifty-five farms in Fujian province, China. Samples were homogenated then subjected to three cycles of freeze–thaw followed by centrifugation at 5000 rpm for 10 min at 4 °C, after which supernatants were collected for DNA extraction. Viral DNA was extracted using the Virus Genomics DNA Isolation Kit (Tianlong Biotech, Suzhou, China) following the manufacturer’s instructions. PCR with pair specific primers (Table 1) was used for detecting PCV3, PCV2, CSFV, and PRRSV [20,21]. PCR conditions were as follows: for each sample 12.5 μL of 2× Taq Master mix (Vazyme Biotech, Shanghai, China), 9.5 μL of double distilled water (ddH2O), 1 μL of template DNA, 0.4 nmol/L forward and 0.4 nmol/L reverse primer were combined. Thermocycler conditions were 95 °C for 5 min, followed by 35 cycles of denaturation at 95 °C for 30 s, annealing at 59 °C for 30 s, and extension at 72 °C for 30 s, with a final extension at 72 °C for 10 min. Samples were held at 4 °C until further use. Complete genomes were amplified using the Phanta Max Super-Fidelity DNA polymerase (Vazyme Biotech, Shanghai, China) with the same amplification conditions, subjected to 1% agarose gel electrophoresis, then sent to Tsingke (Nanjing, China) for sequencing. Sequences were assembled using the BioEdit software [22].

### 2.2. Sequence Collection, Alignment, and Phylogenetic Analysis

Three hundred and six complete PCV3 genomes, collected between 1996 and 2019 from different countries, were downloaded from GenBank at the National Center for Biotechnology Information (https://www.ncbi.nlm.nih.gov/). All sequences were separated by Rep and Cap according coding region, then the coding area was spliced together (Supplementary Table S1). MUSCLE [23] was used to align sequences, and manual adjustments were made to the alignment using MEGA7.0 [24]. After poor quality sequences were removed, 272 reference sequences remained. The best fit nucleotide substitution model was selected using ModelFind in IQ-tree [25]. The maximum likelihood (ML) tree was reconstructed using RAxML (version 2.8.10) [26]. For nucleotide substitution, we used the General Time Reversible parameter plus the GAMMA distribution model (GTR + G) pre-estimated, with 1000 bootstrap replicates. To estimate the time elapsed since the most recent common ancestor and the rate of PCV3 evolution, we constructed a maximum clade credibility (MCC) tree using Markov chain Monte Carlo (MCMC) methods, using BEAST (v1.8.4) [27] and the Cap protein sequences. The substitution model was set to GTR + G with a relaxed molecular clock (lognormal), and the tree prior was coalescent (Bayesian skyline) according to model comparison built in Tracer (http://tree.bio.ed.ac.uk/software/tracer/). The total chain length was 1 × 10^8^, with sampling every 10,000 steps. Two independent runs were performed and then combined using LogCombiner. The final MCC tree was annotated using TreeAnnotator and visualized using Figtree.

### 2.3. Selection Model Analysis

The ML tree based on full-length sequences (spliced ORF1 and ORF2 coding region) was uploaded to DataMonkey (www.datamonkey.org) to estimate branching and sites under selection. Selected sites were identified using four algorithms: single-likelihood ancestor counting (SLAC), mixed effects model of evolution (MEME), fixed effects likelihood (FEL), and fast unconstrained Bayesian approximation (FUBAR). Positively selected branches were identified using the adaptive branch-site REL test for episodic diversification (aBSREL) [28,29,30,31,32]. A site was considered to be under positive selection only if it satisfied at least two algorithms (*p* < 0.1 in SLAC, *p* < 0.05 in FEL and MEME, *p* > 0.9 in FUBAR). Cap sequences from strains PCV3a and PCV3b were used to predict protein structures in I-TASSER (https://zhanglab.ccmb.med.umich.edu/I-TASSER/) [33]. Structures were visualized using PyMoL.

## 3. Results

### 3.1. Characterization of PCV3 Strains Circulating in Fujian

Seven of the 127 samples were positive for PCV3 (positive rate of 5.51%), these samples are henceforth referred to as PCV3-CN-FJ1-2018, PCV3-CN-FJ2-2018, PCV3-CN-FJ22-2018, PCV3-CN-FJ27-2018, PCV3-CN-FJ33-2018, PCV3-CN-FJ37-2018, and PCV3-CN-FJ65-2018 (Supplementary Table S2). It should be noted that these seven samples were negative for PCV2, CSFV and PRRSV. All the PCV3 genomes we sequenced here were 2000 bp. Sequencing analysis of the full-length genomes, and Rep and Cap genes revealed maximum nucleotide sequence similarities amongst each other of 98.85–99.80%, 99.00–100% and 98.60–99.80% respectively, and to reference strains from GenBank of 91.24–99.90%, 98.2–100% and 96.7–100% respectively. Maximum amino acid sequence similarities for Rep and Cap were 99.3–100% and 98.1–100% within the strains, and compared to reference strains were 97.2–100% and 96.7–100% (Table 2), respectively. The 24th amino acid of the Cap protein of PCV3 is a key amino acid that distinguishes PCV3a from PCV3b; this amino acid is valine on the capsids of PCV3-CN-FJ2-2018, PCV3-CN-FJ27-2018 and PCV3-CN-FJ37-2018, and alanine on the capsids of PCV3-CN-FJ1-2018, PCV3-CN-FJ22-2018, PCV3-CN-FJ33-2018, and PCV3-CN-FJ65-2018.

### 3.2. Phylogenetic and Evolution Analysis of PCV3 in China and Worldwide

**Figure 1:**
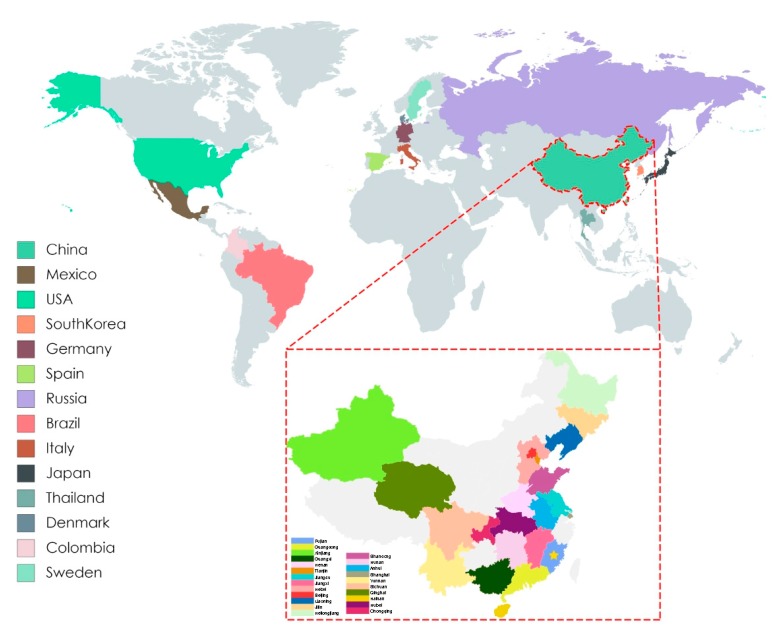
Map of PCV3 positive farms by country, and by province in China. Colored rectangles correspond to indicated PCV3 positive country and Chinese provinces. The yellow star indicates Fujian province.

**Figure 2:**
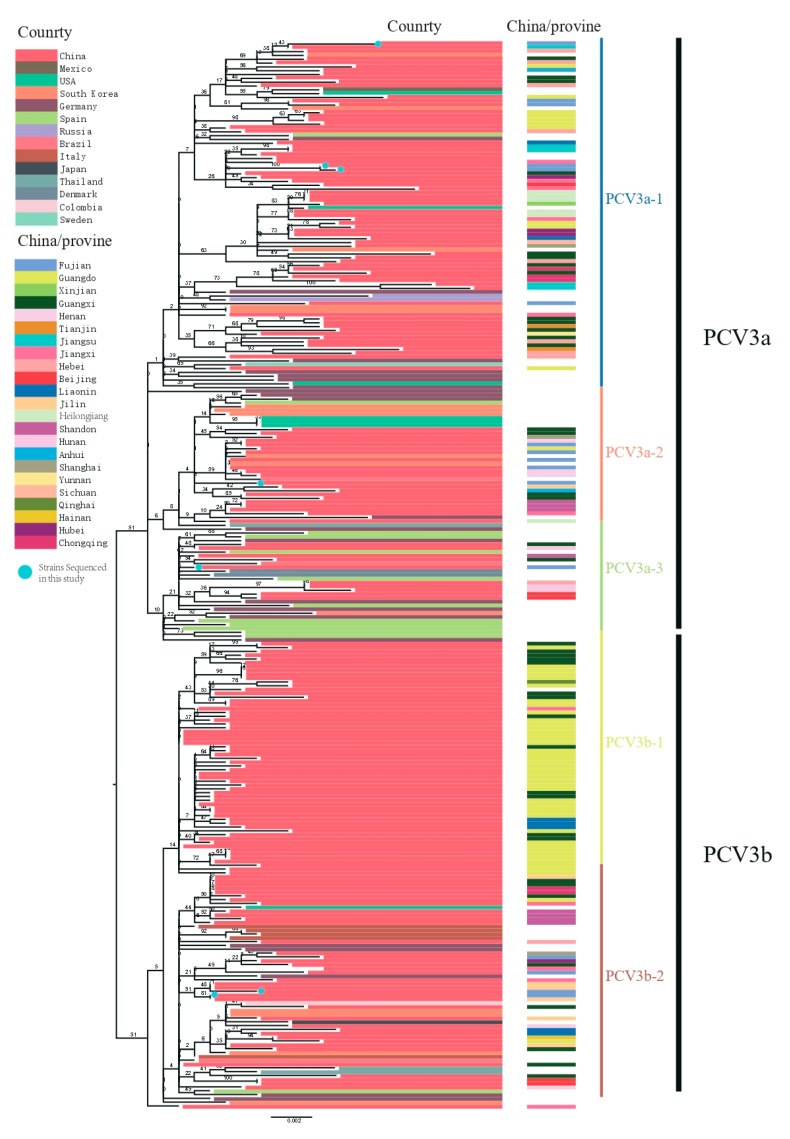
Maximum likelihood trees based on full-length PCV3 sequences. Trees were reconstructed using RAxML (Version 8.4.10) with the general time reversible plus GAMMA (GTR + G) distribution substitution model (1000 bootstraps). Blue dots represent sequenced strains from this study. Colored lines indicate country or region of origin.

**Figure 3:**
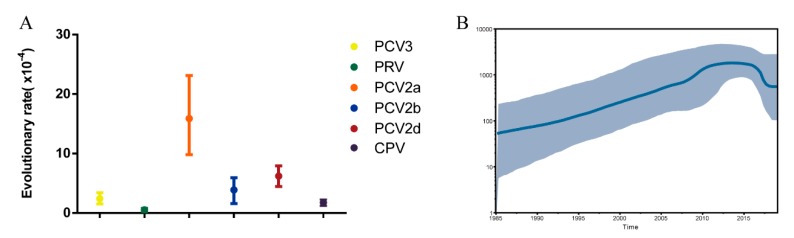
(**A**) Evolutionary rate for the PCV3 Cap gene. The evolutionary rate was estimated using BEAST (v1.8.4) with the GTR + G model and a relaxed lognormal molecular clock. The tree prior was set at coalescent: Bayesian skyline, and total chain length was 1 × 10^8^ with sampling every 10,000 steps. (**B**) Bayesian skyline plot for the PCV3 Cap gene. The mean genetic diversity through time (*N_e_t*) for the PCV3 ORF2 gene is shown by the black boldface line, while the 95% highest probability density (HPD) of *N_e_t* is plotted on the *y*-axis (grey region).

After removal of poor-quality data from an initial set of 306 PCV3 sequences obtained from GenBank, 272 reference sequences from the 14 countries remained (Figure 1). We used these, along with PCV3 sequences generated in our study, to reconstruct phylogenetic trees. Based on the ML tree (Figure 2), PCV3 can be classified into two main clades, PCV3a and PCV3b. At a higher level of resolution, PCV3a can be divided into PCV3a-1, PCV3a-2, and PCV3a-3, while PCV3b is comprised of PCV3b-1 and PCV3b-2. Globally, PCV3a strains outnumber PCV3b strains. Similarly, of the sequences from the PCV3 strains we isolated, five of seven were classified as PCV3a and two as PCV3b. From the tree, we found that the strains we isolated in Fujian were PCV3a-1, PCV3a-2, PCV3a-3 and PCV3b-2. In addition, these strains were close to the strains found in Jilin, Guangxi, Jiangxi, and Jiangsu provinces. The tMRCA was estimated to be 1954.25 with 95% highest probability density (HPD) between 1940.70 and 1984.90, while the evolutionary rate was estimated to be 2.41 × 10^−4^ with 95% HPD between 1.53 × 10^−4^ and 3.40 × 10^−4^ (Figure 3A). 

The scaled effective population size (*Neτ*) for PCV3 was also estimated. Figure 3B shows that the population size increased from 1985 to 2012, then stabilized until 2016. From 2016 to 2019, the population size declined slightly but was still at a high level (about 5 × 10^2^).

### 3.3. Selection and Amino Acid Function Analysis

**Figure 4:**
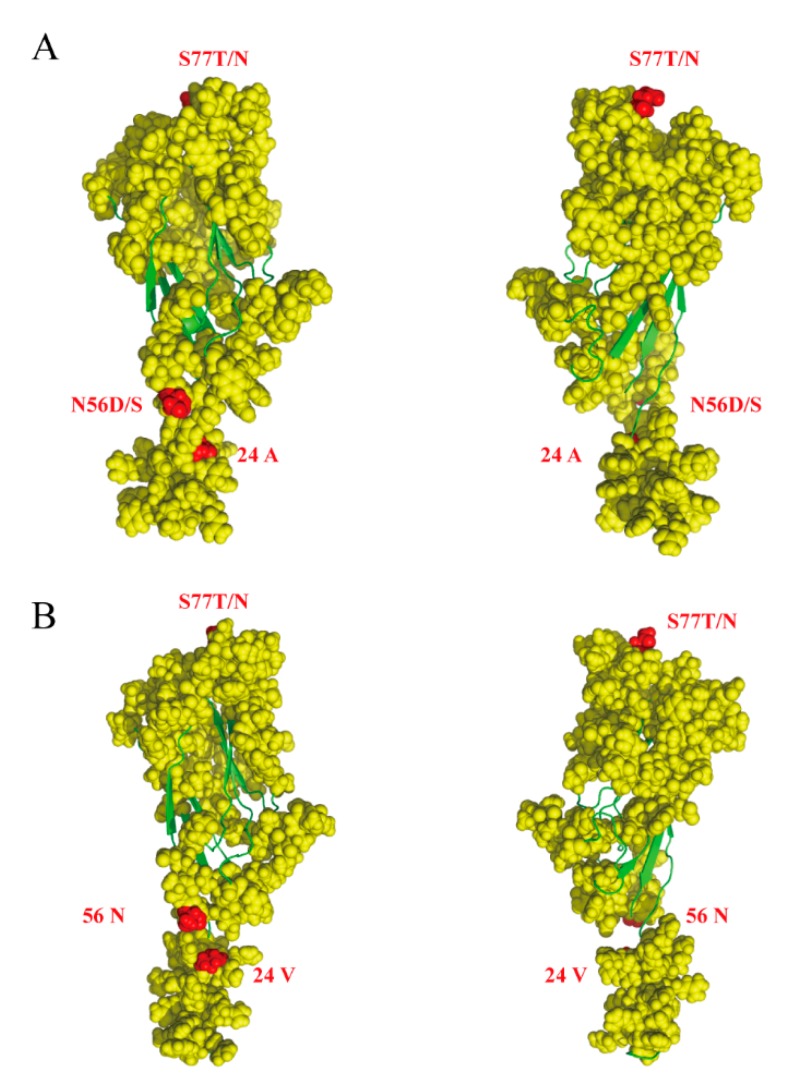
Predicted structure of PCV3 Cap protein. Cap protein structure was predicted using I-TASSER (https://zhanglab.ccmb.med.umich.edu/I-TASSER/). The yellow filled circles indicate the epitope of PCV3 cap protein, the red filled circles indicate amino acids subjected to positive selection, and green lines indicate α-helixes and β-folds. (**A**) PCV3a (MF318452:PCV3-BJ-2), (**B**) PCV3b (KX458235:2164).

Selective pressure analysis demonstrated a clear dominance of sites under negative pressure both in the Rep and Cap proteins [34]. We detected four positively selected amino acid sites, one at residue 122 in the Rep protein, and three at residues 24, 56, and 77 in Cap (Table 3). Residues 122 in Rep and 24 in Cap were detected by all methods (SLAC, FEL, FUBAR and MEME). In contrast, residues 56 and 77 were detected only by FEL and FUBAR. However, there was no positively selected branch in the PCV3 ML tree. Interestingly, several positively selected sites were associated with functional activities. Cap residues 24, 56, and 77 were in the epitope region and were classified as positively selected sites by Li et al. and Sun et al. [7,9] (Figure 4). Residue 122 in Rep was also described as positively selected by Li et al.

## 4. Discussion

The identification of PCV3, a new porcine circovirus resembling the significantly pathogenic PCV2, has raised a great interest in its evolution and epidemiology. In China, the number of emerging swine viruses has increased in the last years [7,35] raising two pressing questions. (i) Are swine novel hosts to these viruses? (ii) What is the epidemic character of PCV3 in China? Although PCV3 strains from most parts of China have been well studied, there is no systematic analysis of the prevalence and evolutionary characteristics of strains in eastern China, especially in Fujian Province. Fujian is a large aquaculture province with a subtropical monsoon climate that provides favorable conditions for the prevalence and evolution of disease.

Although some evidence supports an association between PCV3 infection and clinical disease [6], contradictory reports have been published [36], and more extensive studies are needed. Fu et al., 2018 found that in southern China 22.3% of samples were co-infected with PCV2. Ku et al., 2017 found that 45.4% of PCV3 positive samples were also positive for PCV2, while Zhang et al., 2018 found co-infection of PCV2 and PCV3 in 6.8% of the 265 clinical samples screened in that study. In a survey of samples from Shandong province we found 59.4% were infected with PCV3 alone and 39.3% were co-infected with PCV2 [37]. In this study, we found that all the samples from swine with respiratory symptoms were positive for PCV3 only. Although there are some people think PCV3 is suspicious of the pathogenicity of the herd [38], research on PCV3 pathogenicity should be further studied. Additionally, there are differing views of the phylogenetic analysis of PCV3; we found that PCV3 can be divided into two clades: PCV3a, PCV3b, and five sub-clades PCV3a-1, PCV3a-2, PCV3a-3, PCV3b-1, and PCV3b-2. This agrees with the report by Li et al. [7], but not fully with Ouyang et al., who are using ORF2 gene for analysis [8]. PCV3a is the most wide spread genotype in Fujian province consisting of PCV3a-1, PC3a-2 and PCV3a-3. Meanwhile, PCV3b-2but not PCV3b-1 was also found in Fujian province. Thus, in Fujian province, PCV3 has a high genetic diversity, in addition, the tMRCA of PCV3 is estimated later than in Saraiva et al. [39]. From previous important studies [7], it is known that an alanine at amino acid number 24 distinguishes PCV3a from PCV3b, which has a valine at that site. In our study the strain PCV3-CN-FJ27-2018 had a valine at position 24 but it belongs to the PCV3a group. This prompts us to propose that PCV3 subtypes might recombine with each other during infections, similar to PCV2. After MCMC analysis we found that the evolutionary rate of PCV3 exceeds that of canine parvovirus or porcine pseudorabies virus but is similar to PCV2b and PCV2d [40,41], this result is similar to that reported by Li et al. [7]. However, based on more reference sequences than used in the studies of Li et al., the tMRCA of PCV3 is earlier than reported in those studies. The estimated *Neτ* value of PCV3 is greater than that of Middle East respiratory syndrome coronavirus. We expect scaled effective population size *Neτ* to follow I/2β, where β is the equilibrium rate of transmission and I is the equilibrium number of infections. Although the duration of infection time of PCV3 in swine is unknown, given the large population size, its true value should also be similar to or greater than the estimated value.

Additionally, we found some amino acids on the antigen epitope that have been subject to positive selection during evolution. Similar to previous reports, amino acid 24 and 77 were found to be subjected to positive selection [7,8], however, amino acid 56 was first found in the Cap protein. Our results indicate the action of the host immune response in shaping PCV3 evolution. Swine with respiratory symptoms were positive only for PCV3, which may indicate that PCV3 has gradually adapted to swine and even become pathogenic to swine.

Although the number of sequences in PCV3 is increasing, we found that the analysis results are very similar to those reported previously after removal of poor quality sequences [7]. Pcv3 is still one of the DNA viruses with the highest evolutionary rate. In addition, it is of great concern that the inaccuracy of early samples’ epidemiological results may be caused by many laboratory pollutions. This situation will greatly misleading the evolution rate analysis of certain viruses, especially for new emerging viruses, so the evolutionary dynamics of PCV3 deserve further study. Our study has increased the information on the genetics and molecular characteristics of PCV3. These results will contribute to the evaluation of the relevance of PCV3 to the swine industry, and to the planning of effective control strategies in China.

## Figures and Tables

**Table 1:** Primers used in this study.

	Primers	Amplicon Length (bp)
PCV3-D-F	ACTTAGAGAACGGACTTGTAACGAA	649
PCV3-D-R	AAATGAGACACAGAGCTATATTCAG	
PCV3-1-F	ATTATGGATGCTCCTCATCGTG	553
PCV3-1-R	CATCTTCTCCGCAACTTCAGTC	
PCV3-2-F	GACTGAAGTTGCGGAGAAGATG	789
PCV3-2-R	CGGCACGAAAGAAGTTTGGATT	
PCV3-3-F	CCCACATGCGAGGGCGTTTACC	895
PCV3-4-R	CGAGGCCGCTTCATCATCCACT	
PCV2-D-F	AGAAGCTCTCTATCGGAG	569
PCV2-D-R	AAGGTTGAATTCTGGCCC	
CSFV-D-F	TAGGGTGGACGGGTGTCATAGAGT	566
CSFV-D-R	AAGCATATATTGCTGGAAGTAGCT	
PRRSV-D-F	GCCTCGTGTTGGGTGGCAGAA	532
PRRSV-D-R	CGCCCTAATTGAATAGGTGACTT	

Classical swine fever virus (CSFV), porcine reproductive and respiratory syndrome virus (PRRSV).

**Table 2:** Maximum nucleotide and amino acid sequence divergence of Porcine Circovirus 3 (PCV3).

Isolated Strains	Sequence Similarity among Isolated Strains	Sequence Similarity between Isolated Strains and Reference Strain
Rep gene (nt)	99.0–100%	98.2–100%
Rep gene (aa)	99.3–100%	97.2–100%
Cap gene (nt)	98.6–99.8%	97.8–100%
Cap gene (aa)	98.1–100%	96.7–100%
Full-length (nt)	98.85–99.80%	97.26–99.90%

Replication (Rep), Capsid (Cap), amino acid (aa), nucleotide (nt).

**Table 3:** Selection analysis of full-length PCV3.

AA	FEL		SLAC		FUBAR		MEME	
	dN-dS	*p*-Value	dN-dS	*p*-Value	dN-dS	Post.Pro	w+	*p*-Value
122	6.515	**0.002**	13.8	**0.00217**	11.731	**0.999**	>100	**0**
320(24)	2.784	**0.027**	6.29	0.0585	4.93	**0.993**	>100	**0.02**
373(77)	3.44	**0.039**	5.02	0.285	4.164	**0.982**	>100	0.1
352(56)	3.739	0.055	5.66	0.265	5.578	**0.987**	>100	0.12

## Supplementary Materials

The following are available online at https://www.mdpi.com/1999-4915/11/9/786/s1, Table S1: Reference sequences information used in this study, Table S2: Sequences information sequenced in this study.
