# Supplementary material for: Evolution and Genetic Diversity of Porcine Circovirus 3 in China
Source: Viruses. 2019 Aug 27;11(9):786. doi: 10.3390/v11090786 (PMC6783837; doi:10.3390/v11090786)
Supplement: Supplementary file 1 [file viruses-11-00786-s001.pdf]

Supplementary Table S1. Reference sequences information used in this study.

accession number	isolate name	host	Country	collection date
NC-031753	29160	swine	USA-NorthCarolina	2015
MK454953	GD-HZ01/2017	pig	China	11-2017
MK454952	FJ-PM02/2018	pig	China	09-2018
MK454951	FJ-PM01/2018	pig	China	09-2018
MK340753	PCV3/CN/Xinjiang-12/2018	porcine	China	07-2018
MK142773	GZH-043	swine	China	2018
MK142772	GD-060	swine	China	2017
MK142771	FJ-024	swine	China	2016
MK095625	CH/GX/2275A/2018	swine	China	08-2018
MK095624	CH/GX/2092A/2018	swine	China	04-2018
MK095623	CH/GX/2051A/2018	swine	China	03-2018
MK095622	CH/GX/2031A/2018	swine	China	03-2018
MK095621	CH/GX/1963A/2018	swine	China	01-2018
MK095620	CH/GX/1948/2018	swine	China	01-2018
MH916639	PCV3/Guangxi-CZ/05	dog	China-Guangxi	16-05-2017
MH916638	PCV3/Guangxi-GL/04	dog	China-Guangxi	16-05-2017
MH916636	PCV3/Guangxi-NN/02	dog	China-Guangxi	16-05-2017
MH916635	PCV3/Guangxi-BH/01	dog	China-Guangxi	16-05-2017
MH823221	CH/GX/2248/2018	swine	China-Guangxi	08-2018
MH823220	CH/GX/2246/2018	swine	China-Guangxi	08-2018
MH823219	CH/GX/2242/2018	swine	China-Guangxi	08-2018
MH699985	PCV3-wb/Br/RS	wildboar	Brazil	
MH683051	PCV3/AY/Henan-06/2018	porcine	China	2018
MH607133	PCV3/CH/HB/XY/2018	pig	China	02-2018
MH579747	2018	Wild-boar	Spain	11-02-2018
MH579746	2017	Wild-boar	Spain	26-02-2017
MH579745	2016	Wild-boar	Spain	19-11-2016
MH579743	2014	Wild-boar	Spain	01-03-2014
MH579742	2013	Wild-boar	Spain	26-10-2013
MH579741	2012	Wild-boar	Spain	02-03-2012
MH579740	2011	Wild-boar	Spain	01-01-2011
MH579739	2010	Wild-boar	Spain	05-02-2010
MH579738	2007	Wild-boar	Spain	01-01-2007
MH579737	2006	Wild-boar	Spain	21-01-2006
MH579736	2005	Wild-boar	Spain	06-02-2005
MH558676	1987/2012	swine	Spain	2012
MH547276	PCV3/CH/TianJin-1/2018	swine	China	03-2018
MH522791	TJ-1701	swine	China	01-2017
MH520669	PCV3/GDBH2-2018replication-associated	swine	China	02-2018
MH491030	PCV3/GDLP-2018	swine	China	02-2018
MH491029	PCV3/GD512-2018	swine	China	04-2018
MH491028	PCV3/GDYT2-2017	swine	China	10-2017
MH491027	PCV3/GDYT1-2017	swine	China	08-2017
MH491026	PCV3/GDSG2-2017	swine	China	06-2017
MH491025	PCV3/GDSG1-2017	swine	China	02-2017
MH491024	PCV3/GDCA-2018	swine	China	04-2018
MH491023	PCV3/GDMH2-2018	swine	China	03-2018
MH491022	PCV3/GDMH1-2018	swine	China	03-2018
MH491021	PCV3/GDJW-2018	swine	China	02-2018

MH491020	PCV3/GDHL3-2017	swine	China	12-2017
MH491019	PCV3/GDHL2-2017	swine	China	05-2017
MH491018	PCV3/GDHL1-2017	swine	China	02-2017
MH491017	PCV3/GDDH2-2018	swine	China	03-2018
MH491016	PCV3/GDDH1-2018	swine	China	01-2018
MH445394	Nanjing-BALB-C4	mouse	China	2018
MH445393	Nanjing-BALB-C2	mouse	China	2018
MH410564	PCV3-CN-Jiangxi0114-2016	Sus scrofa	China	14-01-2016
MH410563	PCV3-CN-Hebei0613-2016	Sus scrofa	China	13-06-2016
MH410562	PCV3-CN-Guangxi0610-2015	Sus scrofa	China	10-06-2015
MH410561	PCV3-CN-Beijing0103-2016	Sus scrofa	China	03-01-2016
MH367850	PCV3/CN/GDZW/2017	Sus scrofa	China	2017
MH367849	PCV3/CN/GDST/2017	Sus scrofa	China	2017
MH367848	PCV3/CN/GDHY/2017	Sus scrofa	China	2017
MH367847	PCV3/CN/GDGL2/2017	Sus scrofa	China	2017
MH367846	PCV3/CN/GDGL1/2017	Sus scrofa	China	2017
MH367845	PCV3/CN/GDCC/2017	Sus scrofa	China	2017
MH327785	COL/Cundinamarca2/2018	Porcine	Colombia	2018
MH286898	PCV3-China/DB-1/2017	pig	China	2017
MH277119	PCV3-CN2018LN-5	Sus scrofa	China	2018
MH277118	PCV3-CN2018LN-4	Sus scrofa	China	2018
MH277117	PCV3-CN2018LN-3	Sus scrofa	China	2018
MH277116	PCV3-CN2018LN-2	Sus scrofa	China	2018
MH277115	PCV3-CN2018LN-1	Sus scrofa	China	2018
MH277114	PCV3-CN2018JL-3	Sus scrofa	China	2018
MH277113	PCV3-CN2018JL-2	Sus scrofa	China	2018
MH277112	PCV3-CN2018JL-1	Sus scrofa	China	2018
MH277111	PCV3-CN2018HLG-5	Sus scrofa	China	2018
MH277110	PCV3-CN2018HLG-4	Sus scrofa	China	2018
MH277109	PCV3-CN2018HLG-3	Sus scrofa	China	2018
MH277108	PCV3-CN2018HLG-2	Sus scrofa	China	2018
MH277107	PCV3-CN2018HLG-1	Sus scrofa	China	2018
MH229786	L1513	pig	Thailand	17-05-2017
MH192340	PCV3/MEX/GTO/01/2017	porcine	Mexico	02-07-2017
MH177453	CN/Liaoning-2017	pig	China	03-2017
MH121060	SD17-36	pig	China	30-03-2017
MH107164	PCV3/Shandong-04/2016	bovine	China	2016
MH107163	PCV3/Shandong-03/2016	bovine	China	2016
MH107162	PCV3/Shandong-02/2016	bovine	China	2016
MH107161	PCV3/Shandong-01/2016	bovine	China	2016
MH101645	PCV3/CH/HB/HD/2017	swine	China	11-2017
MH018246	PCV3-HBYX1707	swine	China	07-2017
MG947596	SD	porcine	China	2017
MG897494	PCV3-China/GD-EP/2017	pig	China	01-08-2017
MG897493	PCV3-China/GX-NN-1/2017	pig	China	01-08-2017
MG897490	PCV3-China/GD-SG-PL/2017	pig	China	01-08-2017
MG897489	PCV3-China/GD-SH-4/2017	pig	China	01-08-2017
MG897488	PCV3-China/GD-SH-1/2017	pig	China	01-08-2017
MG897487	PCV3-China/GD-SH-2/2017	pig	China	01-08-2017
MG897486	PCV3-China/GX-WZ-1/2017	pig	China	01-08-2017
MG897485	PCV3-China/GD-ZQ-1/2017	pig	China	01-08-2017
MG897484	PCV3-China/GD-FK-1/2017	pig	China	01-08-2017
MG897483	PCV3-China/GD-HS-DL/2017	pig	China	01-08-2017
MG897480	PCV3-China/GD-HZ-02/2017	pig	China	01-08-2017
MG897479	PCV3-China/GD-HZ-lm/2017	pig	China	01-08-2017

MG897478	PCV3-China/HuN-CS/2017	pig	China	01-08-2017
MG897477	PCV3-China/GD-KP/2017	pig	China	01-08-2017
MG897476	PCV3-China/GD-MM-01/2017	pig	China	01-08-2017
MG897474	PCV3-China/GD-GZ-ZC/2017	pig	China	01-08-2017
MG897473	PCV3-China/GX-NN-2/2017	pig	China	01-08-2017
MG870097	PCV3-China/JL16-38	porcine	China	
MG870096	PCV3-China/JL16-8	porcine	China	
MG870095	PCV3-China/JL17-42	porcine	China	
MG868946	PCV3-AH-201706	swine	China	2017
MG868945	PCV3-SH-201705	swine	China	2017
MG868944	PCV3-LNSY-201705	swine	China	2017
MG868943	PCV3-JSDF-201704	swine	China	2017
MG868942	PCV3-JXXY-201704	swine	China	2017
MG868941	PCV3-HBWH-201703	swine	China	2017
MG868940	PCV3-JSXY-201701	swine	China	2017
MG860486	Henan-ZZ-2016	Swine	China	2016-03-10
MG778698	PCV3CNBeijing-32017SJYH	pig	China	04-04-2017
MG765473	Porcinecircovirus3	Sus scrofa	Sweden	2004
MG727540	PCV3/CH/HB/SJZ/2017	swine	China	08-2017
MG727539	PCV3/CH/HB/CZ-1/2017	swine	China	08-2017
MG727538	PCV3/CH/HB/BD/2017	swine	China	08-2017
MG727537	PCV3/CH/HB/CZ-2/2017	swine	China	08-2017
MG696866	2017-JS2229		China	15-03-2017
MG679917	PCV3-RU/SM17	Domestic pig	Russia	2017
MG679916	PCV3-RU/TY17	Domestic pig	Russia	2017
MG650176	YN3-1996	Domestic pig	China	1996
MG650175	SC6-1998	Domestic pig	China	1998
MG650174	GX16-1998	Domestic pig	China	1998
MG650173	GX15-1998	Domestic pig	China	1998
MG650172	GX11-1998	Domestic pig	China	1998
MG564175	NWHUN2	pig	China	2016
MG564174	NWHEB21	pig	China	2016
MG550107	CH/GX/1776D/2017	Swine	China-Guangxi	16-09-2017
MG546667	PCV3/CN/BJ-YH2016	pig	China	12-2016
MG372492	PCV3/CN/Henan/1/2016	swine	China	05-2016
MG372491	PCV3/CN/Henan/2/2016	swine	China	05-2016
MG372489	PCV3/CN/Hunan/1/2013	swine	China	07-2013
MG372487	PCV3/CN/Hunan/2/2013	swine	China	07-2013
MG372486	PCV3/CN/Qinghai/2016	swine	China	08-2016
MG372485	PCV3/CN/Anhui/2016	swine	China	11-2016
MG372484	PCV3/CN/Fujian/2016	swine	China	05-2016
MG372483	PCV3/CN/Heilongjiang/2017	swine	China	05-2017
MG310152	PCV3/Thailand/PB01/17	pig	Thailand	07-2017
MG253684	Hainan/2017	porcine	China	20-09-2017
MG253683	GX-920/2017	porcine	China	20-09-2017
MG253682	GD-YF/2017	porcine	China	20-09-2017
MG253681	GD-MZ/2017	porcine	China	16-08-2017
MG253680	GD-JM	porcine	China	16-08-2017
MG253679	GD-GZ/2017	porcine	China	16-09-2017
MG253678	GD-FS/2017	porcine	China	12-09-2017
MG250187	PCV3/GXFC2017-12	Sus scrofa	China	2017
MG250186	PCV3/GXFC2017-7	Sus scrofa	China	2017
MG250185	PCV3/GXFC2017-11	Sus scrofa	China	2017
MG250184	PCV3/GXFC2017-10	Sus scrofa	China	2017
MG250183	PCV3/GXFC2017-9	Sus scrofa	China	2017

MG250182	PCV3/GXFC2017-8	Sus scrofa	China	2017
MG250181	PCV3/GXGP2017-6	Sus scrofa	China	2017
MG250180	PCV3/GXGG2017-5	Sus scrofa	China	2017
MG250179	PCV3/GXNN2016-4	Sus scrofa	China	2016
MG250178	PCV3/GXYL-2009-3	Sus scrofa	China	2009
MG250177	PCV3/GXYL2009-2	Sus scrofa	China	2009
MG250176	PCV3/GXYL2009-1	Sus scrofa	China	2017
MG014385	DE31.17	swine	Germany	2015
MG014384	DE28.12	swine	Germany	2015
MG014383	DE17.20	swine	Germany	2015
MG014382	DE15.19	swine	Germany	2015
MG014381	DE14.15	swine	Germany	2015
MG014380	DE12.19	swine	Germany	2015
MG014379	DE6.1	swine	Germany	2015
MG014378	DE5.15	swine	Germany	2015
MG014377	DE2.8	swine	Germany	2015
MG014376	DE55.1	swine	Germany	2015
MG014375	DE53.8	swine	Germany	2015
MG014374	DE52.18	swine	Germany	2015
MG014373	DE48.7	swine	Germany	2015
MG014372	DE41.16	swine	Germany	2015
MG014371	DE34.5	swine	Germany	2015
MG014370	DE27.16	swine	Germany	2015
MG014369	DE26.17	swine	Germany	2015
MG014367	DE19.15	swine	Germany	2015
MG014366	DE18.2	swine	Germany	2015
MG014365	DE13.20	swine	Germany	2015
MG014363	DE4.3	swine	Germany	2015
MG014362	DE3.7	swine	Germany	2015
MF805724	4332-7-Denmark-2017	Swine	Denmark	2017
MF805723	4332-5-Denmark-2017	Swine	Denmark	2017
MF805722	4289-Italy-2016	Swine	Italy	2016
MF805721	32941-Italy-2016	Swine	Italy	2016
MF805720	737-8-Spain-2017	Swine	Spain	2017
MF805719	1621-Italy-2017	Swine	Italy	2017
MF677841	JX-4/CH/2017	pig	China	2017
MF677839	JX-2/CH/2017	pig	China	2017
MF677838	JX-1/CH/2017	pig	China	2017
MF677837	JX-3/CH/2016	pig	China	2016
MF677836	JX-2/CH/2016	pig	China	2016
MF677835	JX-1/CH/2016	pig	China	2016
MF611878	PCK3-1703	swine	SouthKorea	2017
MF611877	PCK3-1702	swine	SouthKorea	2016
MF611876	PCK3-1701	swine	SouthKorea	2016
MF593110	HB-1/2016	pig	China	2016
MF589652	309replication-associatedprotein	Sus scrofa	Thailand	09-2016
MF589107	PCV3/CN/Jiangxi-B1/2017	swine	China	01-2017
MF589106	PCV3/CN/Jiangxi-3/2016	swine	China	02-2016
MF589104	PCV3/CN/Guangdong-MX3/2015	swine	China	08-2015
MF589103	PCV3/CN/Guangdong-HZ4/2015	swine	China	04-2015
MF589102	PCV3/CN/Guangdong-HY1/2016	swine	China	06-2016
MF448445	IH	porcine	SouthKorea-Kyunggido	07-04-2017
MF405277	PCV3/CN/GXHJ2/2017	swine	China	25-04-2017
MF405275	PCV3/CN/GDQG1/2017	swine	China	22-04-2017
MF405274	PCV3/CN/GXLJ2/2017	swine	China	20-04-2017

MF405273	PCV3/CN/GXHJ1/2017	swine	China	15-04-2017
MF405272	PCV3/CN/GDBL1/2017	swine	China	12-04-2017
MF405271	PCV3/CN/GDSJ1/2017	swine	China	07-03-2017
MF318453	PCV3-BJ-1-2016	swine	China	2016
MF318452	PCV3-BJ-2-2015	swine	China	2015
MF318450	PCV3-Hebei-XJ-2015	swine	China	2015
MF318449	PCV3-Hebei-BD-2015	swine	China	2015
MF318448	PCV3-Hebei-HD-2016	swine	China	2016
MF162299	PCV3-IT/MN2017	pig	Italy	03-2017
MF162298	PCV3-IT/CO2017	pig	Italy	03-2017
MF155643	PCV3-China/GX2016-3	pig	China-Guangxiprovince	
MF155642	PCV3-Chian/GX2016-2	pig	China-Guangxiprovince	
MF155641	PCV3-China/GX2016-1	pig	China-Guangxiprovince	
MF079254	PCV3-BR/RS/8	pig	Brazil	2016
MF079253	PCV3-BR/RS/6	pig	Brazil	2016
MF069252	PCV3-CN/FuJian-420-2017	pig	China	2017
MF069116	PCV3/CN/GDHE2/2016	swine	China	15-12-2016
MF069115	PCV3/CN/GDLC1/2016	swine	China	07-12-2016
MF063071	16R927/2016	pig	SouthKorea	2016
MF063070	P1705SCYC/2017	pig	SouthKorea	2017
MA820822	JP2018535251-A/1-PORCINECIRCOVIRUSTYPE3IMMUNOGENIC			
LC383841	Porcinecircovirus3KGS2098-1/2016DNA	Sus scrofadomesticus	Japan-Kagoshima	2016-09-29
KY996345	PCV3/KU-1609	pig	SouthKorea	2016
KY996344	PCV3/KU-1608	pig	SouthKorea	2016
KY996343	PCV3/KU-1607	pig	SouthKorea	2016
KY996342	PCV3/KU-1606	pig	SouthKorea	2016
KY996341	PCV3/KU-1605	pig	SouthKorea	2016
KY996340	PCV3/KU-1604	pig	SouthKorea	2016
KY996339	PCV3/KU-1603	pig	SouthKorea	2016
KY996338	PCV3/KU-1602	pig	SouthKorea	2016
KY996337	PCV3/KU-1601	pig	SouthKorea	2016
KY924472	PCV3-CN/FuJian-512-2016	pig	China	2016
KY865243	CHN-Shanghai-0708-2016	pig	China	07-2016
KY865242	CHN-Shanghai-0706-2016	pig	China	07-2016
KY778777	PCV3/CN/Shandong-2/201703	pig	China	2017
KY778776	PCV3/CN/Shandong-1/201703	pig	China	2017
KY753913	CNGD-2	swine	China-Guangdong	2016
KY753912	CNFJ-1	swine	China-Fujian	2016
KY753911	CNGD-1	swine	China-Guangdong	2016
KY421348	PCV3-CHN/CC2016	swine	China-JilinChangchun	2016
KY421347	PCV3-CHN/GD2016	swine	China-Guangdong	2016
KY418606	PCV3-China/GD2016	swine	China	18-12-2016
KY354039	CN/Hubei-618/2016	porcine	China	2016
KY354038	CN/Hubei-610/2016	porcine	China	2016
KY075994	PCV3/CN/Chongqing-156/2016	porcine	China	2016
KY075993	PCV3/CN/Chongqing-155/2016	porcine	China	2016
KY075992	PCV3/CN/Chongqing-150/2016	porcine	China	2016
KY075991	PCV3/CN/Chongqing-148/2016	porcine	China	2016
KY075990	PCV3/CN/Chongqing-147/2016	porcine	China	2016
KY075989	PCV3/CN/Jiangxi-62/2016	porcine	China	2016
KY075988	PCV3/CN/Henan-13/2016	porcine	China	2016
KY075987	PCV3/CN/Fujian-12/2016	porcine	China	2016
KY075986	PCV3/CN/Fujian-5/2016	porcine	China	2016
KX966193	PCV3-US/SD2016	pig	USA	2016
KX898030	PCV3-US/MN2016		USA	2016

KX778720	PCV3-US/MO2015	pig	USA	2015
KX458235	2164	swine	USA-Oklahoma	2015
KT869077	29160	swine	USA-NorthCarolina	2015

Supplementary Table S2. Sequences information sequenced in this study.

accession number	isolate name	host	Country	collection date
MN075128	FJ1	swine	China:Fujian	2018
MN075129	FJ2	swine	China:Fujian	2018
MN075130	FJ22	swine	China:Fujian	2018
MN075131	FJ27	swine	China:Fujian	2018
MN075132	FJ33	swine	China:Fujian	2018
MN075133	FJ37	swine	China:Fujian	2018
MN075134	FJ65	swine	China:Fujian	2018
